# Elusive super-hard B_6_C accessible through the laser-floating zone method

**DOI:** 10.1038/s41598-019-49985-2

**Published:** 2019-09-16

**Authors:** Bibi Malmal Moshtaghioun, Francisco L. Cumbrera, Diego Gómez-García, Jose I. Peña

**Affiliations:** 1 Instituto de Ciencia de Materiales de Aragón, CSIC-Universidad de Zaragoza, campus Río Ebro, 50018 Zaragoza, Spain; 2 Instituto de Ciencia de Materiales de Sevilla, Departamento de Fisica de la Materia Condensada, CSIC-Universidad de Sevilla, 41092 Sevilla, Spain

**Keywords:** Ceramics, Design, synthesis and processing

## Abstract

Boron carbide is among the most promising ceramic materials nowadays: their mechanical properties are outstanding, and they open potential critical applications in near future. Since sinterability is the most critical drawback to this goal, innovative and competitive sintering procedures are attractive research topics in the science and technology of this carbide. This work reports the pioneer use of the laser-floating zone technique with this carbide. Crystallographic, microstructural and mechanical characterization of the so-prepared samples is carefully analysed. One unexpected output is the fabrication of a B_6_C composite when critical conditions of growth rate are adopted. Since this is one of the hardest materials in Nature and it is achievable only under extremely high pressures and temperatures in hot-pressing, the use of this technique offers a promising alternative for the fabrication. Hardness and elastic modulus of this material reached to 52 GPa and 600 GPa respectively, which is close to theoretical predictions reported in literature.

## Introduction

Boron carbide (B_4_C) ceramics have been widely considered as high performant ceramic materials thanks to its ultra-high hardness, low density and other promising properties like high elastic modulus, wear resistance and melting point, good thermal stability and somewhat low material cost. This is the reason why boron carbide is the first potential candidate for several structural applications like tribo-component ceramics, nuclear radiation shields, ceramic armours for ballistic protection of both personnel and vehicles^1–3^. It is quite known that the mechanical properties of boron carbide which make it demanding ceramics strongly depend on porosity, composition, microstructure and fabrication process^4–19^. Purity is an attractive goal, because pure ceramics normally exhibit optimal mechanical properties and can be studied rigorously due to their reproducibility. In this sense, it is also important from a basic viewpoint because their intrinsic properties (unaltered by additives) are attainable. Therefore, fabrication of pure and near full-dense boron carbide ceramic with retaining its unique properties is the main challenge nowadays. As a main limitation, densification of B_4_C is rather difficult in the pure state by conventional solid-state sintering methods like pressureless sintering or hot-pressing: in fact, very extreme sintering conditions are required to this purpose^1,2^. Until now, electric field sintering techniques have been found to be the promising method in general, especially for these hard to-sinter ceramics^7–14^. However, the industrial upscale of the technology is limited due to the reduced size of the samples attainable and the material heterogeneities in large samples and especially the finished cost does not sound reasonable.

The laser floating zone (LFZ) technique is a well-established crystal growth method in materials research, able to produce very high melting point materials with extremely high purity and low cost compared to other advanced techniques. In this method, ceramics grown from melt are found to be near fully dense with fine and homogeneous microstructure. In addition to this, the final pieces present higher potential regarding their mechanical properties^20,21^. This method is found as a powerful one to grow a long list of oxide ceramics and their composites and eutectics^20,21^. Recently, boride and carbide eutectic ceramics, specially boron carbide eutectic systems, have been fabricated with this technique with the aim of fabricating hard and high-melting point B_4_C based ceramics with the properties close to those of pure boron carbide^22–24^. However, there is no attempt to grow a pure and monolithic polycrystalline boron carbide ceramic. This option would thus seem to in principle be more desirable with a view to retaining its intrinsic ultra-high hardness, a largely desired objective for these advanced ceramics.

In this study, we aim to investigate firstly the possibility of fabrication of pure B_4_C ceramics by laser floating zone technique. Secondly the comprehensive understanding of the microstructure, composition and some preliminary mechanical properties of grown boron carbide with variable growth rates are performed.

## Results and Discussion

**Figure 1:**
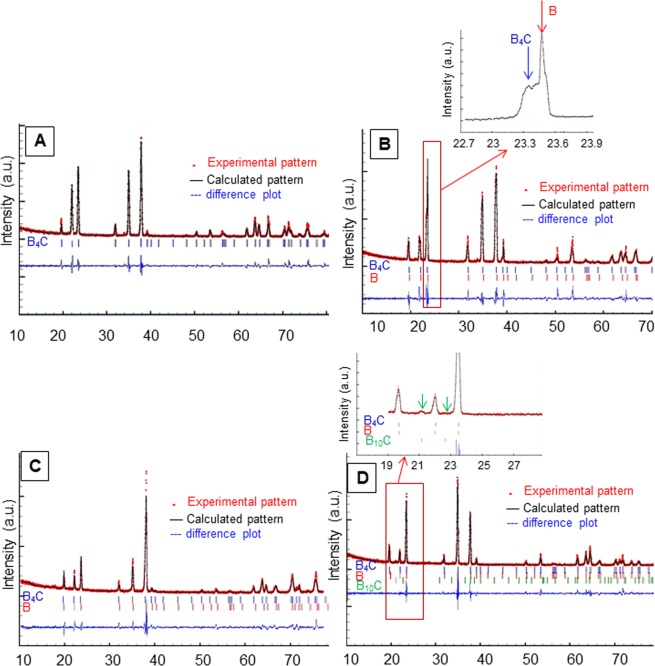
XRD patterns of the boron carbide ceramics grown at (**A**) 150, (**B**) 300, (**C**) 500 and (**D**) 750 mm/h. An example of the overlapped peaks of boron carbide and metallic boron are shown at ~23° in inset. (**B**) The position of B_10_C peaks are shown by green arrows in inset (**D**).

The idealized structure of boron carbide is commonly described as a rhombohedral unit cell that contains one icosahedral B_12_ unit and one C-B-C chain. The B_12_ units are composed of crystallographically distinct boron atoms, named as equatorial and polar, in a D_3d_ environment. However, this is an ideal archetype. Due to the wide range of substitutional carbon composition which can be accommodated in the lattice, significant deviations from the idealized model can be found^25^. In our case, Rietveld refinements of the boron carbide ceramics grown at different velocities are provided in Fig. 1. The crystallographic results after iterative fitting for all samples are displayed in Table 1. Our results show that growth velocity has strong effects on the boron carbide structure and stoichiometry. For low growth rate of 150 mm/h, the structure fits into a model of combination of 73% B_11_C_E_ icosahedra and 27% B_12_ icosahedra with chains of C-B-C or C–C. (E stands for “equatorial” position and is a vacancy). 12% vacancies are found in the boron positions of chains and the final stoichiometry is estimated as B_4.45_C, which is close to the simple structural formula B_4_C (Fig. 1A). However, a slight increase of boron content and carbon loss is detected at this growth rate. At higher growth velocities, the carbon loss becomes evident and the ceramic approaches to a boron-rich boron carbide structure with the additional presence of some elementary boron. The peaks of this element exhibit a tiny angle difference and overlap with those of boron carbide (Fig. 1B-inset). More precisely, for 300 mm/h growth rate, 51% B_11_C_E_ icosahedra and 49% B_12_ icosahedra with chains of C-B-C are the outputs provided by Rietveld analysis (Fig. 1B) which is formulated as B_4.98_C (~B_5_C). 22 wt% of elementary boron with the same space group of R $\bar{3}$ m and rhombohedral cell structure is also detected. At 500 mm/h growth rate, 17% B_11_C_E_ icosahedra and 83% B_12_ icosahedra are predicted, with chains composed of C-B-C giving rise to a stoichiometry of B_5.91_C (~B_6_C). At this growth rate, the amount of elementary boron increases to 32 wt% (Fig. 1C). This trend of shifting to higher boron-rich boron carbide phase breaks down when the growth rate reaches 750 mm/h. This fact can be inferred from the systematic increase of the lattice parameter with the growth rate until 500 mm/h, which drops for a growth rate of 750 mm/h (Table 1). This is a logical finding due to the difference in the atomic radius between carbon and boron: thus boron-rich boron carbides are expected to have slightly expanded lattice, as reported in literature^17,18,25^. For 750 mm/h growth rate, the structure fits into a model of again high percentage of B_11_C_P_ icosahedra (80%) and B_12_ icosahedra (20%). Now the suffix “P” stands for “polar” position (see Table 1). In this case, the chains are randomly distributed into either C-B-C (81.3%), C-B-B (13.2%) or C-C-C (4.3%) (Fig. 1D). The stoichiometry approaches to B_4.36_C which again is close to the common B_4_C formula. The amount of elementary boron is decreased to ~11 wt%. In addition to this, the presence of an ultra-boron-rich boron carbide with formula B_10_C as a minor second phase is detected. The peaks related to this phase are shown in Fig. 1D-inset and the amount is estimated around 1.31 wt%. In general, no trace of boron oxide or residual carbon is found in any pattern. We tried to find the new theoretically predicted B_14_C but our fitting is not compatible with their structural parameters and symmetry^26^.

**Table 1:** Structural parameters for boron carbide ceramics grown at different velocities obtained by Rietveld refinement. Occupation factors are referred to a single icosahedron.

Growth velocity (mm/h)		150	300	500	750
Space group		R $\bar{{\bf{3}}}$ m	R $\bar{{\bf{3}}}$ m	R $\bar{{\bf{3}}}$ m	R $\bar{{\bf{3}}}$ m
Lattice parameters	a (Å)	5.5998 (6)	5.6087 (2)	5.6247 (13)	5.6097 (4)
	c (Å)	12.0733 (15)	12.0945 (6)	12.1243 (26)	12.0924 (8)
Occupancy	B1_E_ 18 h (equatorial position)	5.27	5.49	5.83	6.00
	B2_P_ 18 h (Polar position)	6.00	6.00	6.00	4.80
	B3 3b (chain position, center)	0.88	1.00	1.00	0.95
	C1 6c (chain position, extremes)	2.00	2.00	2.00	1.85
	C2 18 h (equatorial position)	0.73	0.51	0.17	—
	C3 18 h (Polar position)	—	—	—	1.2
	B4 6c (chain position, extremes)	—	—	—	0.15
	C4 3b (chain position, center)	—	—	—	0.05
Composition		73% (B_11_C)_E_ and 27% (B_12_). The chain is C-B-C with 12% vacancies in chain centers.	51% (B_11_C)_E_ and 49% (B_12_). The chain is C-B-C. No vacancies detected.	17% (B_11_C)_E_ and 83% (B_12_). The chain is C-B-C. No vacancies detected	80% (B_11_C)_P_ and 20% (B_12_). The chains are: C-B-C (81.3%), C-B-B (13.2%) and C-C-C (4.3%). 5% vacancies detected.
Stoichiometry		B_4.45_C	B_4.98_C	B_5.91_C	B_4.36_C
Other phases		—	22.04 wt% of boron	32.42 wt% of boron	10.79 wt% of boron and 1.31 wt% B_10_C
Preferential orientation factor [0001]		0.949	0.921	0.796	0.779
${{\rm{\chi }}}_{{\rm{red}}}^{2}$		4.14	3.66	2.25	2.18

**Figure 2:**
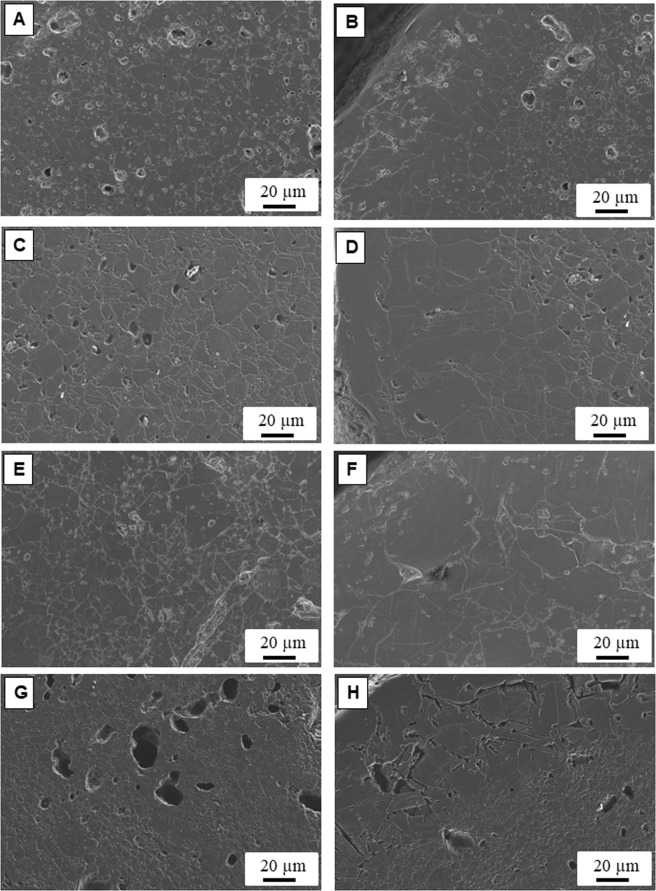
Representative FE-SEM micrographs of the boron carbide ceramics grown at (**A**) 150 mm/h-centre of the rod, (**B**) 150 mm/h-border of the rod (**C**) 300 mm/h-centre of the rod, (**D**) 300 mm/h-border of the rod, (**E**) 500 mm/h-centre of the rod (**F**) 500 mm/h-border of the rod (**G**) 750 mm/h-centre of the rod and (**D**) 750 mm/h-border of the rod.

Figure 2 compares representative FE-SEM images of the B_4_C ceramics grown at different velocities; the summary of microstructural features is listed in Table 2. At a low growth rate of 150 mm/h that corresponds to the less boron-rich sample (100 wt% B_4.45_C), equiaxial grains with an average grain size of ~12 µm are found in the centre of the grown rod (Fig. 2A) and there is a border of ~130–150 µm with coarser grains of 37 µm in size (Fig. 2B). The presence of pores is observed in the interior of the grown rod at this growth rate. Contrary to what is expected for directional solidification of ceramics, i.e. the higher growth rate, the finer the microstructure, is not valid in our case and here the microstructure is strongly dependent on boron carbide composition. At the growth rate of 300 mm/h, average grain size in the centre of grown rod reaches to ~19 µm (Fig. 2C), suggesting important grain growth under the influence of the presence of a boron-rich boron carbide of ~B_5_C with 20 wt% of elementary boron. The grain size in the border grows up to ~61 µm that is a substantial grain growth as well (Fig. 2D). Finer pores are observed in the interior of the grown rod at this growth rate. It is reported before^17–19^, that very low porosity and a remarkable grain growth occurs in boron-rich boron carbide ceramics because of the improvement of bulk diffusion in the presence of additional boron. This tendency continues for higher growth rate of 500 mm/h, thus providing grown rods richer in boron content (~B_6_C) and higher amount of elementary boron (~32 wt%) and porous-free and average grain sizes in the centre and border estimated to be around 23 µm and 98 µm, respectively (Fig. 2E,F). This is the first time that this elusive phase of boron carbide can be straightforwardly fabricated. Within the uncertainty of our experimental set-up (±50 mm/h), 500 mm/hr is the optimal growth rate for B_6_C obtention.

**Table 2:** Growth rates, compositions, microstructural features and mechanical response by nanoindentation of boron carbide ceramics prepared in this study.

Growth velocity (mm/h)	Composition	Grain size in Center			Ave. grain size in border (µm)	Thickness of border (µm)	Hardness (GPa)	Elastic modulus (GPa)
		Ave. (µm)	Min. (µm)	Max. (µm)				
150	100 wt% B_4.45_C	11.7 ± 8	2.4	33.4	37 ± 20	~130–150	36 ± 6	483 ± 40
300	77.96 wt% B_4.98_C-20.04 wt%B	18.8 ± 11	4.9	54.0	61 ± 30	~150–230	39 ± 8	516 ± 50
500	67.85 wt% B_5.91_C-32.42 wt%B	22.6 ± 12	4.6	57.7	98 ± 30	~200–300	52 ± 9	601 ± 60
750	87.90 wt% B_4.36_C-1.31 wt% B_10_C-10.79 wt%B	8.9 ± 5	2.8	23.8	40 ± 20	~180–210	33 ± 9	454 ± 50

At higher grown rate of 750 mm/h, the trend breaks down: the average grain size goes down to ~9 µm and ~24 µm in the centre and border of grown rods, respectively (Fig. 2G,H) and larger pores are found in the microstructure. This is consistent with the composition found for this ceramic, which is recovering a carbon-rich boron carbide structure (~B_4.4_C). Therefore, diminished grain growth is expected. However, a 11 wt% of metallic boron and the trace of second phase (~1.3 wt%) with highest boron rich boron carbide structure of B_10_C is detected. In all growth rates, the thickness of border is almost similar (~150–300 µm).

**Figure 3:**
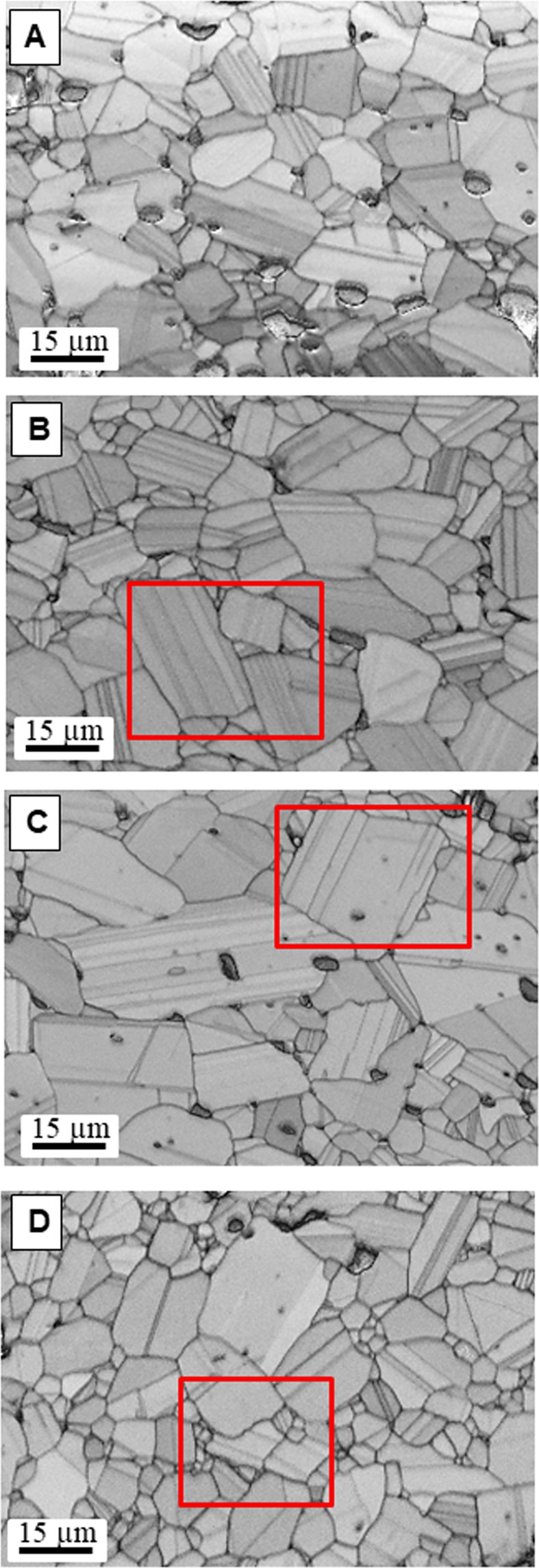
Band contrast map from the EBSD data acquired from boron carbide ceramics grown at (**A**,**B**) 150, (**C**,**D**) 300, (**E**,**F**) 500 and (**G**,**H**) 750 mm/h.

In order to assess the changes in microstructure with growth rate, let us now compare complementary SEM-EBSD results (Fig. 3). In general, for all band contrast maps from EBSD data, the very bright and dark features in the micrographs are pores and shadowing from the grains next to the pores. Planar features/twins are observed in many grains for all velocities. However, there is a higher percentage of planar defects/twins for growth rates of 300 and 500 mm/h which corresponds to boron-rich boron carbide composition (Fig. 3C,E). Our previous results have shown the presence of twins in sintered boron carbide ceramics which controls the kinetics of grain growth^13^. The dominant role of twins on room temperature^27^ and high temperature mechanical behaviour of this ceramic^28,29^ has been proved. More recently, it is reported that boron-rich boron carbides are more disposed to form planar defects during sintering and due at least in part to their lower stacking fault energy which tends to form growth twins^19^. At this regard, it is important to emphasize that the local composition close to twin boundaries can be altered due to the stress field of this. This is a pending problem out of the scope of this work which deserves studying.

**Figure 4:**
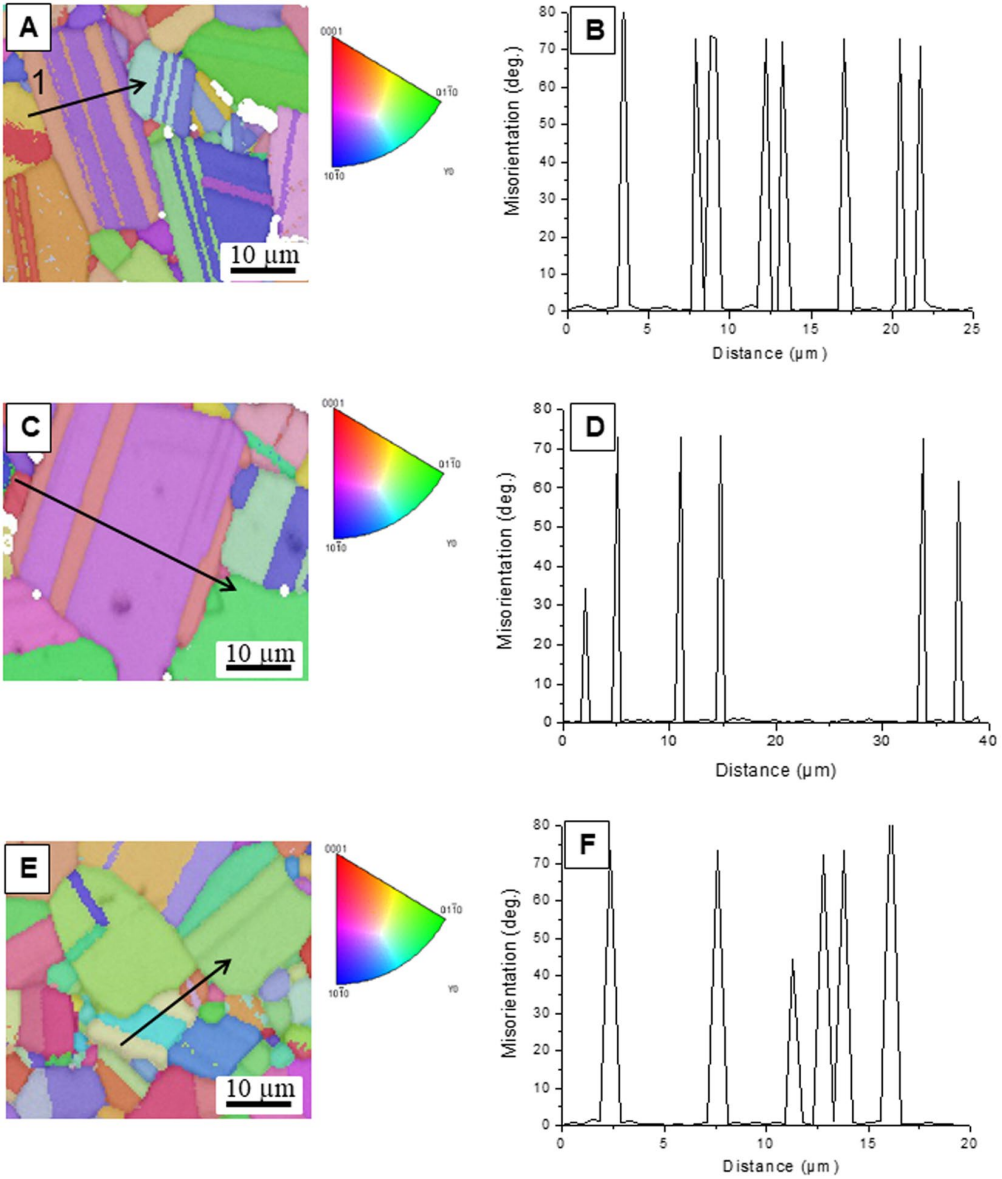
Respectively, orientation map and the measured misorientation across the interfaces shown by black arrows from the area represented by red rectangular in Fig. 3 for boron carbide ceramics grown at (**A**,**B**) 300, (**C**,**D**) 500 and (**E**,**F**) 750 mm/h.

To confirm that the lamella structures observed in the band contrast maps for different velocities and compositions are really twins, the orientations maps from the red rectangular area in Fig. 3 are shown in Fig. 4. The results of misorientation angle across the interfaces for all compositions support that the measured angle for twins is all around 72–73° (Fig. 4B,D,F) that is in good agreement with the known twin relationship in boron carbide^30^. However, the misorientation of interfaces which correspond to regular grain boundaries are lower or higher than this value.

**Figure 5:**
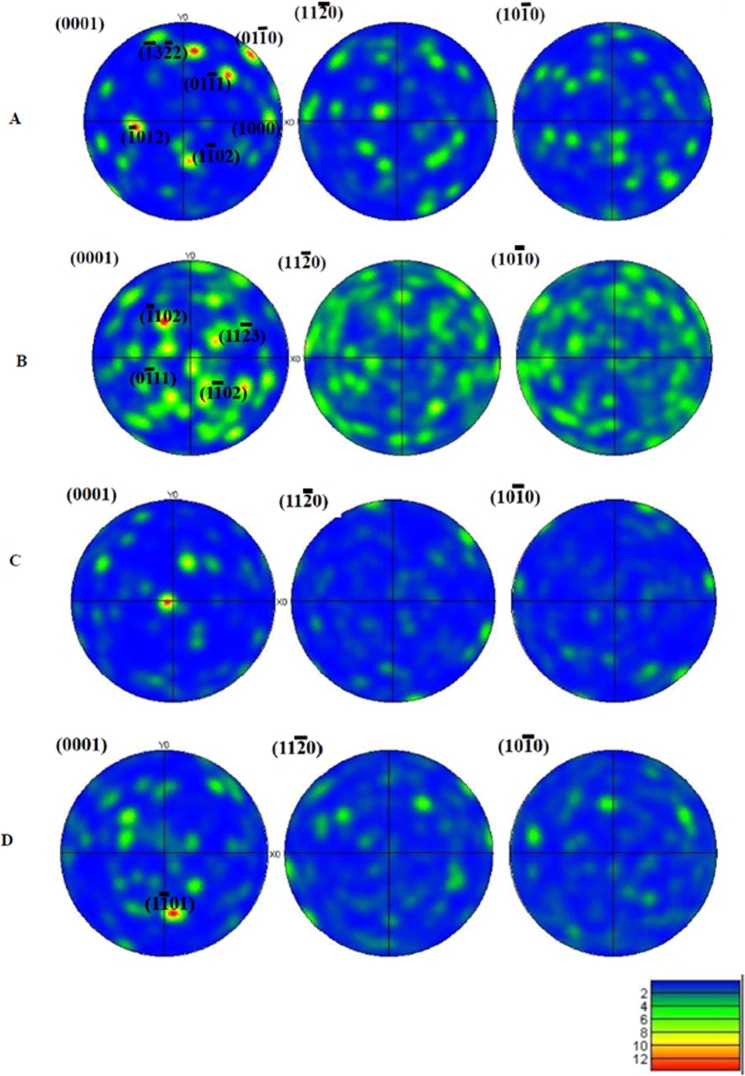
Pole figures of the different samples considered in this study (labelled from **A** to **D**). The (hkil) indexes of poles with a high density are provided in the (0001) stereogram.

The analysis of pole figures (see Fig. 5) shows an increasing tendency to texturing: an inspection of the pole figures centred in (0001) shows that normal of planes are progressively more closed to that direction in the reciprocal lattice. Figure 5A shows the poles of higher density, which have been identified and reported in the (0001) zone axis plot. They correspond mostly to low index planes, i.e. planes de high density. In the case of Fig. 5C, the one corresponding to the B_6_C phase, the texturing is obvious: the longer c axes of grains are aligned along the direction of grown rods, in a columnar growth-type. This increasing tendency correlates with the monotonous increase of the lattice parameters with the growth rate and it breaks down when the growth rate is 750 mm/h and the B_10_C phase is formed. No dominant texture is detected in the zone axes normal to the (0001). This result is congruent with the preferential crystallographic orientations determined by the March-Dollase approach^31^. According to this analysis, displayed in Table 1, the orientation of directions increases with the growth velocities, although it decreases slightly for the specimen grown at the highest velocity.

In terms of mechanical properties, hardness and elastic modulus of boron carbide rods grown at variable velocities measured by nanoindentation using a 250 mN load and the results are listed in Table 2. Since all samples have almost ten or more than ten micro-meter grain size, therefore the grain size dependence of hardness is minimized and here the composition of boron carbide has stronger influence on hardness values. For this reason, nano-indentation tests were performed to find the intrinsic hardness for each composition. The results show that increasing the growth rate to 500 mm/h hardness and elastic modulus are increasing to 52 GPa and 600 GPa, respectively, and later at higher velocity of 750 mm/h both parameters drop again to 33 GPa and 450 GPa, respectively. This behaviour can be explained with the change of composition. It means boron-rich boron carbides which contain higher densities of twins and lower porosity get higher hardness and elastic modulus. Furthermore, elementary boron with rhombohedral cell structure also is well-known to have high hardness of around 40 GPa^32^ which reinforce intrinsic ultra-hard character of boron-rich boron carbide ceramics. High density planar defects have been reported to further improve the hardness of boron-based ceramics^33^ and specifically boron carbide systems^34^. Furthermore, ab-initio calculations have also confirmed that the hardness of boron-rich boron carbide with stoichiometry of B_6_C can reach very high values (48 GPa), much more than conventional B_4_C (32–35 GPa)^35^. Regarding the elastic modulus, our results can be compared with those reported by McClellan^36^ *et al*. in B_5.6_C single crystals grown by optical floating zone. Their results show a very high anisotropic Young’s modulus which varies in-between 420–520 GPa. The isotropic elastic modulus derived by the Voigt, Reuss or Hill approximations from the single crystals elastic constants is found to be 460.07 GPa. They are significantly smaller than our results for this quantity. The difference can be related to the different stoichiometry, or more likely, to the strong strengthening induced by twinning in polycrystals^29^.

Finally, two pieces of consideration must be remarked: the laser-floating zone method, contrary to other successful fabrication methods for boron carbide like SPS, has great practical interest since it can readily be transferred to the ceramics industry due to its potential scalability. On the other hand, this research work opens interesting basic questions: what is the microscopic mechanism driving the formation of such unusual phase? Are there other rare phases accessible through this technique? In the case of boron carbide, one hint which allows a partial response to the first question: according to quantum chemical calculations, boron-rich phases of boron carbide are energetically more stable than those with replacement of boron with carbon^25^. For sure, laser enhances the tendency to removing carbon, although the detailed mechanism for removal is not known. Information of this mechanism will certainly help to answer the second question. This is for sure the objective of further research in the forthcoming future.

## Conclusions

Boron carbide ceramics were grown by the LFZ method with the aim of studying their sinterability, stoichiometry, microstructure and properties. Based on the experimental results and analyses, the following conclusions can be addressed:

1. LFZ is a competitive and efficient technique to fabricate several boron-rich phases of boron carbide ceramics. Moreover, it has been put forth that a careful selection of the processing conditions permits controlling the stoichiometry of this material.
2. A superhard B_6_C can be fabricated under certain experimental conditions determined in this work. The Young modulus and the nanoindentation hardness are by far the largest ever measured in this material, and very close to the theoretical limit predicted in literature.

## Experimental Procedure

The starting materials were commercially available B_4_C powder (Grade HD20, H. C. Starck, Germany with average particle size around 500 nm). Precursor rods of diameters ~1.8–2 mm and up to 5 cm length were prepared by cold isostatic pressing for 5 min at 200 MPa followed by pre-sintering in a tubular furnace (TermoLab TH1700, Portugal) under a flowing atmosphere of ultra-high purity Ar at 1525 °C for 2 h in order to achieve handling strength.

Boron carbide rods were grown by solidification from the melt using the laser-heated floating zone (LFZ) method with a CO_2_ laser^21^. The rods were grown in argon atmosphere and the growth chamber was kept with a slight overpressure of 0.25 bar above ambient pressure in order to avoid the appearance of voids in the solidified rods. A variable growth rate between 150 and 750 mm/h was chosen to evaluate its effect on the composition, stoichiometry, average grain size, hardness of the resulting boron carbide ceramics. A nominal laser output power of ~220 W has been used to maintain a constant feed and very small molten zone. All B_4_C rods were grown with rotation of the pre-sintered ones with 50 rpm and rods of ~1.3 mm was fabricated.

X-ray powder diffraction patterns were recorded with a Philips X’Pert-Pro diffractometer to analyze the structural changes of grown boron carbide ceramics. In order to determine the crystallographic data and accurate compositions of grown samples in growth rates, Rietveld refinement, using the FullProf program was performed^37^. The Rietveld method is a mathematical tool which consists of a least square numerical fitting of all the diffraction data of intensities of a diffractogram assuming one structural model as well as several parameters accounting for the experimental set-up. The method is an iterative protocol in which the researcher introduces as an input the space group, lattice parameters and a model of crystal lattice. In this model the tentative position of vacancies is introduced. This mode permits calculating the structure factor, which ultimately is assessed by minimalization of the residual difference between experimental and theoretical values of the diffraction intensities^38^.

Even though B and C are contiguous in the periodic table, the boron/carbon interchange can be detected unambiguously since it implies the replacement of carbon atoms in 6c by Boron ones in 18 h. In addition to this, the reliability of the detection was assessed thoroughly through PowderCell.

The microstructure was studied in polished cross-sections of grown boron carbide ceramics using the electron images obtained in a field-emission scanning electron microscopy ((FE-SEM) (model Merlin, Carl Zeiss, Germany). The polished surfaces had previously been electro-chemically etched with a solution of 1% KOH. The orientation relationships of grown phases were determined by electron back-scattered diffraction experiments (EBSD). The experiments were performed on polished and non-etched transverse cross-sections using an EBSD system (model HKL from Oxford Instruments, United Kingdom) integrated in a Merlin field-emission scanning electron microscope (SEM) from Carl Zeiss (Germany). The Channel 5 software was used to index the patterns, build up the orientation maps and obtain the pole figures and orientation relationships^39^.

Furthermore, nanoindentation tests (Agilent Technologies G200, USA equipped with a Berkovich indentor) at 250 mN with constant loading rate of 0.5 mN/s were done to measure hardness and elastic modulus from loading-unloading curves. The values of hardness and elastic modulus measured by nanoindentor were obtained by average of 30 indentation tests at different positions to minimize the effect of sample surface roughness and grain orientation.

## Data Availability

All data are available to the interested researchers on request.
